# Vitamin D Receptor Gene Expression and Function in a South African Population: Ethnicity, Vitamin D and *Fok*I

**DOI:** 10.1371/journal.pone.0067663

**Published:** 2013-06-21

**Authors:** Vanessa O′Neill, Furaha Florence Asani, Tamsyn Jacki Jeffery, Donovan Sean Saccone, Liza Bornman

**Affiliations:** Department of Biochemistry, University of Johannesburg, Johannesburg, Gauteng, South Africa; University of Tennessee, United States of America

## Abstract

Polymorphisms of the vitamin D receptor gene (*VDR*) have been associated inconsistently with various diseases, across populations of diverse origin. The T(f) allele of the functional SNP *Fok*I, in exon 2 of *VDR*, results in a longer vitamin D receptor protein (VDR) isoform, proposed to be less active. Genetic association of *VDR* with disease is likely confounded by ethnicity and environmental factors such as plasma 25(OH)D_3_ status. We hypothesized that *VDR* expression, VDR level and transactivation of target genes, *CAMP* and *CYP24A1*, depend on vitamin D, ethnicity and *Fok*I genotype. Healthy volunteers participated in the study (African, n = 40 and White, n = 20). Plasma 25(OH)D_3_ levels were quantified by LC-MS and monocytes cultured, with or without 1,25(OH)_2_D_3_. Gene expression and protein level was quantified using qRT-PCR and flow cytometry, respectively. Mean plasma 25(OH)D_3_ status was normal and not significantly different between ethnicities. Neither 25(OH)D_3_ status nor 1,25(OH)_2_D_3_ supplementation significantly influenced expression or level of VDR. Africans had significantly higher mean VDR protein levels (*P*<0.050), nonetheless transactivated less *CAMP* expression than Whites. Genotyping the *Fok*I polymorphism by pyrosequencing together with HapMap data, showed a significantly higher (*P*<0.050) frequency of the CC genotype in Africans than in Whites. *Fok*I genotype, however, did not influence *VDR* expression or VDR level, but influenced overall transactivation of *CAMP* and 1,25(OH)_2_D_3_-elicited *CYP24A1* induction; the latter, interacting with ethnicity. In conclusion, differential *VDR* expression relates to ethnicity, rather than 25(OH)D_3_ status and *Fok*I genotype. Instead, VDR transactivation of *CAMP* is influenced by *Fok*I genotype and, together with ethnicity, influence 1,25(OH)_2_D_3_-elicited *CYP24A1* expression. Thus, the expression and role of VDR to transactivate target genes is determined not only by genetics, but also by ethnicity and environment involving complex interactions which may confound disease association.

## Introduction

The vitamin D receptor (VDR) is a ligand-activated transcription factor that mediates the genomic actions of vitamin D. These actions involve regulation of calcium homeostasis, cell growth and differentiation, detoxification of xenobiotics, and modulation of adaptive and innate immunity; the latter including activation of monocyte-macrophages [1], [2]. 1,25(OH)_2_D_3_-bound VDR facilitates heterodimerization with the retinoid X receptor (RXR) and binding to vitamin D response elements (VDREs), essential for transcription of VDR-regulated genes. Thus, 1,25(OH)_2_D_3_ availability determines VDR-mediated transactivation of target genes. The genes coding for 1,25(OH)_2_D_3_-catabolizing cytochrome P450 enzyme (*CYP24A1*) and the human cathelicidin antimicrobial protein (*CAMP*) are examples of 1,25(OH)_2_D_3_-regulated target genes. Differential expression of *CYP24A1* and *CAMP* may affect vitamin D status [3] and susceptibility to infectious diseases [4], respectively. Two functional VDREs have been characterized in the promoter of murine *CYP24A1* genes [5], [6] and at least two functional VDREs downstream of the human *CYP24A1* [7]. *CAMP* contains at least one identified VDRE in its promoter region [8], and is induced by 1,25(OH)_2_D_3_ supplementation in primary keratinocytes, monocytes, and neutrophils [9].

Across populations, single nucleotide polymorphisms (SNPs) in the *VDR* have been associated inconsistently with diseases of diverse etiology, including tuberculosis (TB), multiple sclerosis, systemic lupus erythematosus (SLE), cirrhosis and various types of cancer [10]. Among *VDR* SNPs, the functional SNP rs2228570, commonly known as *Fok*I, has been studied extensively. The ancestral f allele (T nucleotide) for this start codon polymorphism, codes for a full length VDR, while the F allele (C nucleotide) results in a three amino acid truncated VDR protein. While it has been shown that the shorter isoform interacts more efficiently with TFIIB [11], [12], reports on the impact of *Fok*I on transactivation are conflicting. Comparing isoforms, Van Etten *et al*. (2007) found no difference in the transactivation mediated by classical DR3-type VDREs [13], while Alimirah *et al*. (2011) showed a 1.8 fold higher transactivation of *CYP24A1* by the shorter variant, compared to the longer isoform [14].

The impact of debilitating variants on VDR function could be exacerbated by vitamin D deficiency or, alternatively, reduced by adequate vitamin D production or intake. For example, Wilkinson *et al*. (2000) observed the TT/Tt genotype of the *VDR Taq*I SNP to be associated with TB in Guajarati Indians living in London, only if vitamin D status was inadequate [15]. The risk of colorectal cancer, the cancer most strongly associated with *VDR*, more than doubles when individuals carrying the ff genotype of *Fok*I consume a low-calcium or low-fat diet, compared to FF genotypes [16]. Thus, vitamin D status influences the impact of *VDR* variants on VDR function and associated disease risk. The widely studied association between vitamin D status and disease supports vitamin D deficiency to be involved in impaired immune function. While latitude and consequent UVB intensity influences vitamin D production in the skin [17], a major determinant of vitamin D status is believed to be skin melanin concentration [18]; with individuals with the darker skin type, VI, having notably less vitamin D than those with white skin type I. Similar to *VDR* variants being associated with disease, vitamin D deficiency have been associated with a higher incidence of TB [19], [20], colorectal cancer [21], [22], [23], cardiovascular disease and SLE [24]. It is uncertain whether the association between vitamin D deficiency and disease prevalence is the cause or effect of disease [20]. Randomized control trials assessing the effect of vitamin D supplementation on disease incidence and prognosis had mixed outcomes. Murdoch *et al*. (2012) observed no impact of vitamin D supplementation on the incidence of upper respiratory tract infections [25]. Gepner *et al*. (2012) observed reduced cardiovascular disease risk in postmenopausal women with vitamin D supplementation [26]. Few studies have, however, evaluated whether interactions between genetic variants in the *VDR* and vitamin D status could confound disease association in diverse populations. In one such a study, Martineau *et al*. (2011) observed an interaction between vitamin D supplementation and the *Taq*I *VDR* polymorphism in TB patients; the tt genotype, but not the Tt/TT, reducing sputum conversion time [27].

We hypothesized that *VDR* expression, VDR level and transactivation of target genes, *CAMP* and *CYP24A1*, depend on a combination of vitamin D, ethnicity and *Fok*I genotype. We assessed the effect of vitamin D, ethnicity and *Fok*I genotype on expression and the functional capabilities of the VDR. Results support that differential VDR expression relates to ethnicity, rather than 25(OH)D_3_ status and *Fok*I genotype. Instead, VDR transactivation of *CAMP* is influenced by *Fok*I genotype which, together with ethnicity, influences 1,25(OH)_2_D_3_-elicited *CYP24A1* expression.

## Materials and Methods

### Participants and sample collection

Participants were healthy blood donors from the South African National Blood Service (SANBS). Ethical clearance was approved for this study by the Ethics Committees of SANBS and the University of Johannesburg, Faculty of Science. After informed written consent, SANBS collected blood from volunteers by venepuncture and prepared buffy coats. The study included only donors of legal donating age (16 and above), therefore consent from the next of kin, caretakers, or guardians on the behalf of the minors/children participants was not required. Buffy coats, tested to be HIV negative, were supplied anonymously within 24 h of venepuncture. Demographics of the study population are summarized in Table 1.

**Table 1:** **Demographics of the study population.**

Demographic parameters	
Number of subjects	60
Sex	
MaleFemale	2931
Ethnicity	
African aWhite	4020
Mean age (range)	35 (17–65)

a African donors belonged to any one of the 4 major ethnic groups living in South Africa. These include the Nguni, Sotho, Shangaan-Tsonga and Venda groups. The Nguni group can be subdivided into Zulu and Xhosa. Donors were collected in the Gauteng province of South Africa, residing in the urban region in and around Johannesburg.

### Plasma vitamin D quantification

Plasma 25-hydroxyvitamin D_3_ (25(OH)D_3_) level is currently the best representative measure of vitamin D status; as 1,25(OH)_2_D_3_ level is under tight control by parathyroid hormone, calcium and phosphorus levels and kept mostly within reference ranges [28]. The levels of 25(OH)D_3_ were quantified in plasma by LC-MS at the Department of Chemical Pathology, Faculty of Health Science, University of Witwatersrand. Plasma 25(OH)D_3_ was extracted according to manufacturer's guidelines using the ClinRep® HPLC Complete 25-OH-Vitamin D_2_/D_3_ Kit (RECIPE, Germany). A commercially available internal standard (RECIPE, Germany) was included and LC-MS was performed using a m/z transition of 401>383 for quantification of 25(OH)D_3_. Serum pools from the Vitamin D External Quality Assessment Scheme (DEQAS, UK) were included in the analysis for quality control purposes.

### Monocyte cultures and treatment

Peripheral blood mononuclear cells (PBMCs) were isolated from the buffy coats using a histopaque-1077® gradient (Sigma Aldrich, St Louis, MO). PBMCs were suspended in tissue culture media containing RPMI (GIBCO, Auckland, New Zealand), 10% FCS, 1% Streptomycin and 1% L-glutamine (Highveld Biological, Johannesburg, South Africa). Cells were seeded on a growth area of 189 cm^2^ per buffy coat and allowed to adhere for 2 h at 37°C, 5% CO_2_. Adhered monocytes were washed, harvested and reseeded at 10×10^6^ cells per culture dish (60 mm diameter). Cultures were left untreated for 16 h after which they were stimulated for 24 h in the presence or absence of 10 nM 1,25(OH)_2_D_3_ (Sigma Aldrich, St Louis, MO). In addition to environmental factors, 25(OH)D_3_ conversion to the active 1,25(OH)_2_D_3_ may be influenced by polymorphisms in, for example, the 1á-hydroxylase gene (*CYP27A1*) [29]. Treating cells with the active 1,25(OH)_2_D_3_ would overcome any genetic variation in this regard and was therefore the metabolite of choice for *in vitro* supplementation.

### qRT-PCR

Expression of *VDR* and its target genes (*CAMP* and *CYP24A1*), were determined by quantitative reverse transcriptase PCR (qRT-PCR). RNA was extracted from monocyte-macrophages with QIAzol® lysis reagent (QIAGEN Sciences, Maryland, USA) according to the manufacturer's guidelines, with the exception that the lysis reagent was increased to 2 ml per 4×10^6^ cells. Extracted RNA was re-dissolved in 25 ìl DEPC-treated water, quantified using Nanodrop spectrophotometry and integrity evaluated using agarose gel electrophoresis. DNA contamination was eliminated using the RQ-1 RNase-free DNase kit (Promega, SA) according to the manufacturer's guidelines. cDNA synthesis was performed using the Tetro cDNA synthesis kit (Bioline, Celtic MolecularDiagnostics, SA). qPCR reactions for each treatment were carried out in duplicate, using Sensimix ™ SYBR No-ROX kit (Bioline, Celtic Diagnostics SA) and the CFX96™ Real-time system, C1000™ Thermal Cycler. Gene normalisation was performed against two stably expressed reference genes: Ubiquitin C (*UBC*) and tyrosine-3-monooxygenase/tryptophan-5-monooxygenase activation protein, zeta polypeptide (*YWHAZ*) [30]. Gene expression was quantified using the comparative C_T_ method according to the MIQE guidelines [31], using inter-run calibrators [32] and qBASE^PLUS^ software [33]. Primer sequences used in this study are listed in Table 2.

**Table 2:** **qRT-PCR primer sequences for target and reference gene amplification.**

Gene	Forward primer	Reverse primer
*VDR*	5′ CTGACCCTGGAGACTTTGAC 3′	5′ TTCCTCTGCACTTCCTCATC 3′
*CAMP*	5′ GCAGTCACCAGAGGATTGTGAC 3′	5′ CACCGCTTCACCAGCCC 3′
*CYP24A1*	5′ ATGAGCACGTTTGGGAGGAT 3′	5′ TGCCAGACCTTGGTGTTGAG 3′
*UBC*	5′ ATTTGGGTCGCGGTTCTTG 3′	5′ TGCCTTGACATTCTCGATGGT 3′
*YWHAZ*	5′ ACTTTTGGTACATTGTGGCTTCAA 3′	5′ CCGCCAGGACAAACCAGTAT 3′

Primer sequences obtained from RTPrimerDB (Bustin *et al*., 2009; http://medgen.ugent.be/rtprimerdb/)

### Flow cytometry

Intracellular VDR protein levels were quantified by flow cytometry in triplicate. Cells (1×10^6^) were permeabilized with 0.2% Triton X-100 (Sigma Aldrich, St Louis, MO) in phosphate buffered saline (PBS). Permeabilized cells were incubated with mouse anti-human IgG_2_ monoclonal antibody against VDR, purchased from Santa Cruz Biotechnologies, Santa Cruz, CA (20 µg/ml 1% BSA/PBS, 30 min). Unbound primary antibody was washed off (0.2% Triton X-100 in PBS) before the cells were labelled with FITC-conjugated goat anti-mouse-IgG_2a_ secondary antibody (8 µg/ml 1% BSA/PBS) purchased from Santa Cruz Biotechnologies, Santa Cruz, CA. Fluorescence was quantified using a BD FACS ARIA™ Flow Cytometer (excitation: 488 nm, emission: 525 nm). Bead-normalized compensation was performed to control for technical variation in fluorescence readings over time [34].

### Genotyping

The *Fok*I polymorphism (rs2228570) in the *VDR* (chr12:48272895, NCBI dbSNP build 137) was genotyped using pyrosequencing. Genomic DNA was extracted from monocytes using the Nucleon™ BACC2 Genomic DNA Extraction kit at 4°C, according to the manufacturer's instructions (GE Healthcare, Buckinghamshire, UK). Extracted DNA was re-dissolved in 50 µl Tris-EDTA buffer (TE, pH 7.4, Sigma Aldrich, St Louis, MO). Genotyping was outsourced to Epigen DX (MA, USA). HapMap population data were obtained from the International HapMap Project, including HapMap Phase I, II and III samples (release 27) from four populations [35]. These populations include individuals from the Centre d′Etude du Polymorphisme Humain (CEPH) collected in Utah, USA, with ancestry from northern and western Europe (CEU; n = 113); Yoruba in Ibadan, Nigeria (YRI; n = 112); Luhya in Webuye, Kenya (LWK; n = 86) and Maasai in Kinyawa, Kenya (MKK; n = 142).

### Statistical Analysis

Statistical analysis was performed using IBM® SPSS® Statistics version 21 for Windows (SPSS Inc., Chicago, Illinois). Gene expression data showed an overall positive skewness and was ln-transformed to obtain normal distribution, meeting the assumption for parametric tests. Two-way ANOVA was used to test whether an interaction exists between treatment and ethnicity or whether these factors have main effects on the data. Pair-wise comparisons of means were computed with the Fisher's least significant difference (LSD) test, with Bonferroni correction for multiple variables. An independent t-test was used to compare plasma 25(OH)D_3_ levels between Africans and Whites. Chi-square analysis of the frequency distribution of genotypes between populations was performed using Microsoft Excel®.

### Data availability

Data presented in this manuscript has been deposited in the Dryad Repository: http://dx.doi.org/10.5061/dryad.12dp5.

## Results

### Ethnicity influences VDR expression and protein level

**Figure 1:**
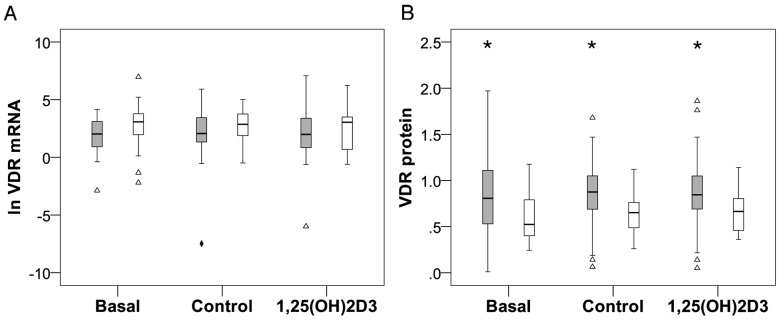
Ethnicity influenced *VDR* mRNA and protein level. *In vitro VDR* expression (A) and protein level (B) were quantified in monocyte-macrophages from healthy Africans (grey, n = 40) and Whites (white, n = 20), using RT-qPCR and flow cytometry respectively. Monocytes were analysed directly after isolation (Basal), or cultured for 24 h with 10 nM 1,25(OH)_2_D_3_ or vehicle control. Box plots show data distribution: boxes illustrate 50% of cases or interquartile range (IQR, 25th to 75th percentile); horizontal lines, median; whiskers, 1.5 IQR from box, or minimum or maximum values if no case has a value in that range. Africans displayed more variance than Whites as illustrated by the distribution IQRs and outliers (Δ, 1.5 to 3 IQR from box) and extreme outlier (♦, >3 IQR from box). Approximately 95% of the data lie between whiskers. Two-way ANOVA showed an overall, significant main effect for ethnicity; Africans having lower *VDR* mRNA (*P*<0.050) but higher protein level (*P*<0.001) compared to Whites. Fisher's LSD test showed a significantly higher mean VDR protein level in Africans compared to Whites under all conditions (* *P*<0.050). *VDR* mRNA data was ln-transformed to meet the assumptions of parametric statistical tests.

To determine whether ethnicity or 1,25(OH)_2_D_3_ supplementation influence *VDR* expression and protein level, primary monocyte-macrophage cultures were established from an African and White population and supplemented *in vitro* with or without 10 nM 1,25(OH)_2_D_3_ for 24 h. *VDR* expression and protein level were quantified and the data was analysed with ethnicity as a fixed factor. Two-way ANOVA revealed a significant main effect of ethnicity on *VDR* mRNA (*P*<0.050) and protein level (*P*<0.001), without treatment interaction (Fig. 1). Fisher's least significant difference (LSD) test showed a significantly higher mean VDR protein level in Africans compared to Whites at basal and control conditions (*P*<0.050) and in the presence of *in vitro* 1,25(OH)_2_D_3_ supplementation (*P* = 0.050; Fig. 1B). *In vitro* 1,25(OH)_2_D_3_ supplementation for 24 h did not significantly alter *VDR* mRNA or protein level compared to the vehicle-treated control irrespective of ethnicity. Post-hoc LSD significance for differences in VDR protein level between Africans and Whites was not maintained after Bonferroni correction.

### VDR-1,25(OH)_2_D_3_ transactivation of target gene CAMP, not CYP24A1, was influenced by ethnicity

**Figure 2:**
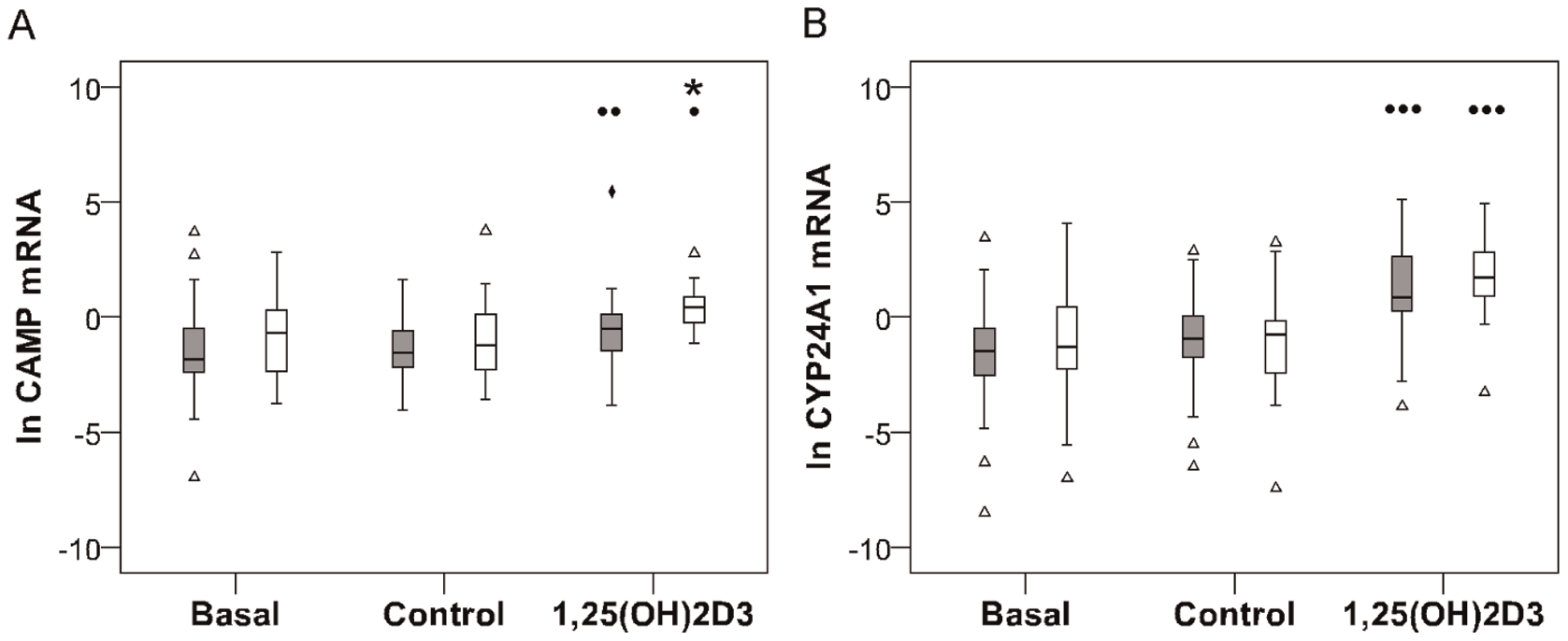
1,25(OH)_2_D_3_-elicited transactivation of target gene *CAMP* by VDR is influenced by ethnicity. Box plots illustrate expression of VDR target genes, *CAMP* (A) and *CYP24A1* (B). Data is differentiated by ethnicity: Africans (grey, n = 40) and Whites (white, n = 20). Gene expression was quantified in monocyte-macrophages from healthy individuals using RT-qPCR. *In vitro* 1,25(OH)_2_D_3_ supplementation significantly induced *CAMP* and *CYP24A1* expression in both Africans and Whites relative to the vehicle control level (*P*<.050, *P*<0.010, P<0.001). Ethnicity had a significant main effect on *CAMP* (*P*<0.010), but not *CYP24A1* mRNA level. Significantly higher mean *CAMP* mRNA level was observed in Whites compared to Africans after 1,25(OH)_2_D_3_ supplementation (* *P*<0.050). *CAMP* and *CYP24A1* mRNA data was ln-transformed to meet the assumptions of parametric statistical analysis. Outliers are defined in legend for Fig. 1.

To evaluate VDR function the mRNA level of VDR target genes, *CAMP* and *CYP24A1*, was quantified in response to *in vitro* 1,25(OH)_2_D_3_ supplementation. *In vitro* 1,25(OH)_2_D_3_ supplementation significantly induced *CAMP* and *CYP24A1* gene expression in both Africans (*CAMP*: *P*<0.010; *CYP24A1*: *P*<0.001) and Whites (*CAMP*: *P*<0.050; *CYP24A1*: *P*<0.001). Ethnicity had a significant main effect on *CAMP* (*P*<0.010), being higher in Whites, but not on *CYP24A1* mRNA level (Fig. 2). The extent of *CAMP* induction by 1,25(OH)_2_D_3_ was ethnicity dependent, as pair-wise comparisons (LSD) showed significantly higher mean 1,25(OH)_2_D_3_-elicited *CAMP* expression in Whites compared to Africans (*P*<0.050). Although the same trend was observed for *CYP24A1* expression, the difference was not significant. The significant difference in *CAMP* levels between Africans and Caucasians was not maintained after Bonferoni correction. Ethnicity-dependent differences in baseline levels of VDR protein (Fig. 1B) prompted the evaluation of transactivation efficiency of VDR (1,25(OH)_2_D_3_-elicited target gene expression level/VDR protein level). In the presence of 10 nM 1,25(OH)_2_D_3_, VDR transactivation efficiency was marginally lower in Africans than Whites for *CAMP* (Africans  = 1.79; Whites  = 2.17) and *CYP24A1* (Africans  = 3.73; Whites  = 4.04) induction.

### The normal 25(OH)D_3_ status in Africans and Whites did not influence VDR expression, protein level or function

**Figure 3:**
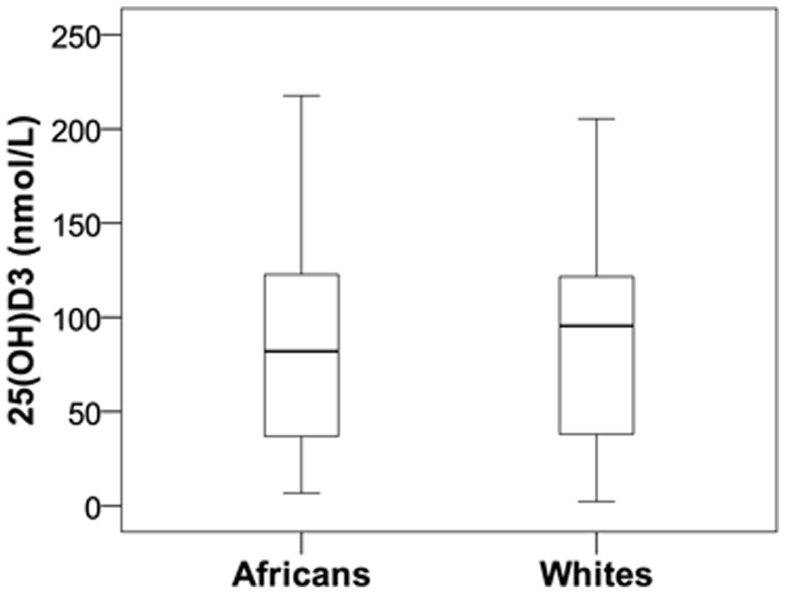
25(OH)D_3_ status of Africans and Whites of the Gauteng Province of South Africa is normal and not significantly different. The mean plasma 25(OH)D_3_ status of Africans (84.4 nmol/L, n = 30) and Whites (92.4 nmol/L, n = 14), quantified using LC-MS, was normal according to the IOM recommendations and not significantly different between the two ethnic groups.

To determine whether variation in the 25(OH)D_3_ status of Africans and Whites contributed to the differential VDR function, plasma 25(OH)D_3_ level was quantified using LC-MS. Overall, the study population had a mean 25(OH)D_3_ status of 87 nmol/L (Fig. 3). Although Whites (92.9 nmol/L) had a slightly higher mean 25(OH)D_3_ status compared to Africans (84.4 nmol/L), the difference was not significant. Neither overall nor condition-specific correlation analysis showed any relation between 25(OH)D_3_ status and *in vitro VDR* expression, VDR level or VDR function in monocyte-macrophages for Africans and Whites combined or in isolation (data not shown).

### The impact of increasing in vitro 1,25(OH)_2_D_3_ supplementation on CAMP expression

**Figure 4:**
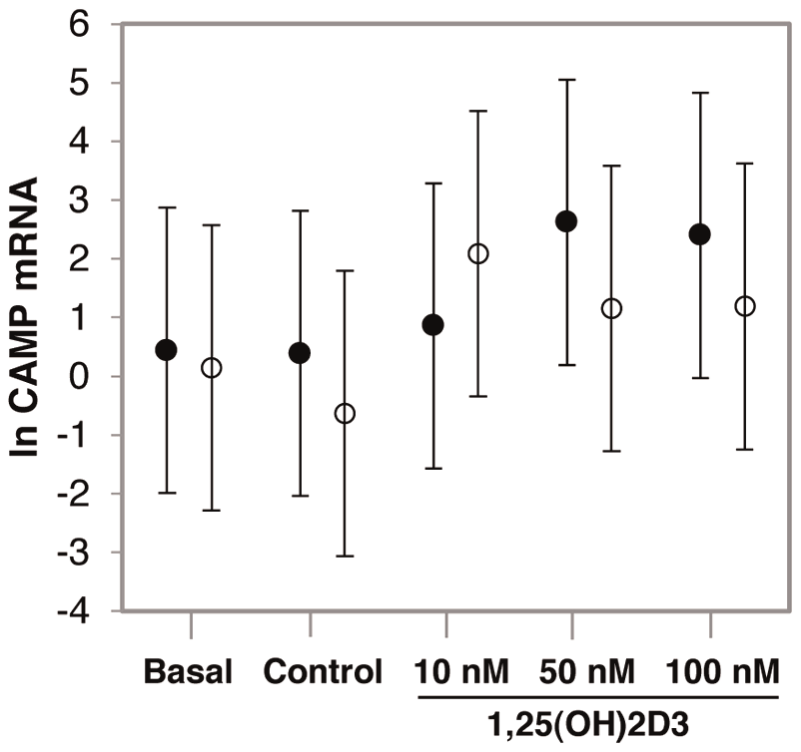
The impact of increasing *in vitro* 1,25(OH)_2_D_3_ supplementation on *CAMP* expression. Error-bar plots illustrate the mean level of *CAMP* expression. Data is differentiated by ethnicity: Africans (black, n = 4) and Whites (white, n = 4). Gene expression was quantified in monocyte-macrophages from healthy individuals using RT-qPCR before (basal) and after 24 h of *in vitro* 1,25(OH)_2_D_3_ supplementation at increasing concentrations (10 nM, 50 nM, and 100 nM). The trend for higher *CAMP* in Whites and lower *CAMP* in Africans at 10 nM, appears to be reversed at higher levels of 1,25(OH)_2_D_3_ supplementation (50 nM and 100 nM). Error bars display the LSD for each data set.

**Figure 5:**
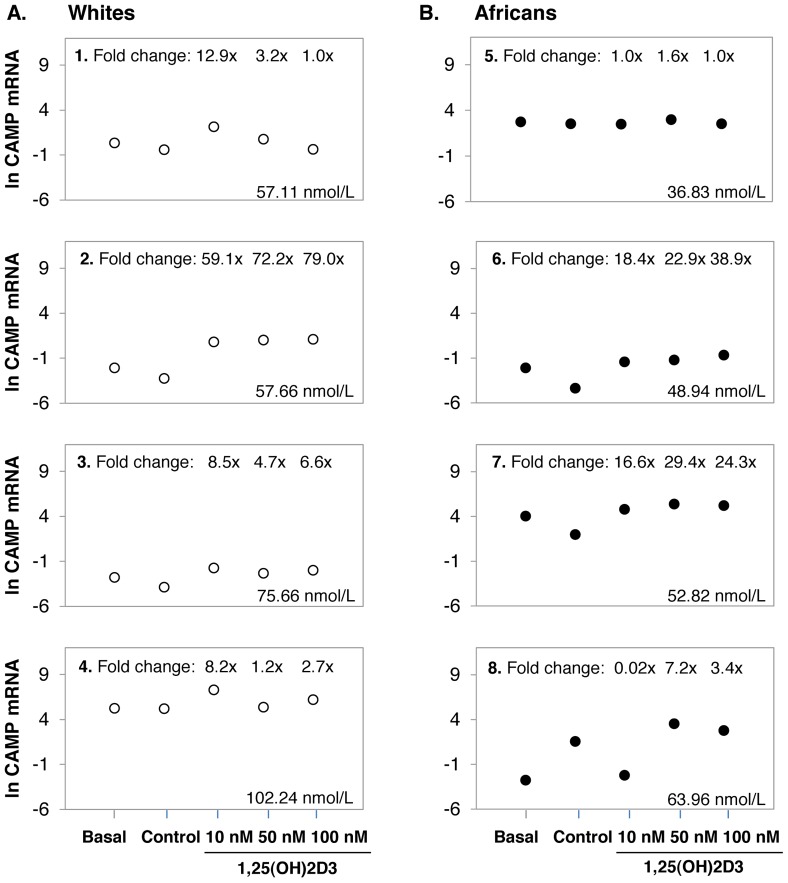
Individual-specific response to increasing *in vitro* 1,25(OH)_2_D_3_ supplementation is 25(OH)D_3_ status-independent. Dots illustrate the mean level of *CAMP* expression per individual for Whites (A, 1–4) and Africans (B, 5–8). The 25(OH)D_3_ status, as well as the 1,25(OH)_2_D_3_-mediated fold change in expression level relative to the control is shown for each individual (untransformed data). Gene expression was quantified in monocyte-macrophages from healthy individuals using RT-qPCR before (basal) and after 24 h of *in vitro* 1,25(OH)_2_D_3_ supplementation at increasing concentrations (10 nM, 50 nM, and 100 nM). Plasma 25(OH)D_3_ status was quantified using LC-MS. A trend towards higher *CAMP* level is present at 10 nM 1,25(OH)_2_D_3_ for Whites, while Africans showed marginally increased *CAMP* expression at 50 nM 1,25(OH)_2_D_3_ concentrations. No trend between plasma 25(OH)D_3_ status and *in vitro* response to increasing concentrations of 1,25(OH)_2_D_3_ is present.

To determine whether the effect of ethnicity on *in vitro VDR* expression, VDR protein level and function would be influenced by increasing concentrations of 1,25(OH)_2_D_3_, a dose response was performed in an independent study in Africans (n = 4) and Whites (n = 4). Considering the mean of only 4 replicates per group, there was no differential response between Africans and Whites, as observed in the larger study, likely due to the notable inter-individual variation in responses, eminent throughout the study (Fig. 4, data not shown for *VDR* and *CYP24A1*). However, the trend for higher *CAMP* in Whites and lower *CAMP* in Africans at 10 nM, appeared to be reversed at higher levels of 1,25(OH)_2_D_3_ supplementation (50 nM and 100 nM).The notable inter-individual variation observed in the larger study prompted us to consider individual responses in the dose response analysis (Fig. 5). In all four White individuals 10 nM 1,25(OH)_2_D_3_ increased *CAMP* expression, which was only true in the case of two of the four African individuals. The two Africans lacking a response at 10 nM (individual 5 and 8) increased *CAMP* expression at 50 nM 1,25(OH)_2_D_3_, but not at 100 nM. In 75% of Whites responding at 10 nM (individual 1, 3 and 4), and 75% of Africans responding at 50 nM (individual 5, 7 and 8), increased 1,25(OH)_2_D_3_ concentration was not necessarily beneficial in terms of *CAMP* expression, and in some cases reduced *CAMP* expression. For the 8 individuals used in the dose-response study, the mean plasma 25(OH)D_3_ status was not significantly different between Africans and Whites and did not correlate with *VDR* mRNA level, VDR protein level or function in any condition.

### *Fok*I genotype distribution differs between Africans and Whites

The CC genotype of the *Fok*I SNP is a functional coding variant in the *VDR* that could potentially contribute to differential VDR function in Africans and Whites. We evaluated the *Fok*I genotype distribution between African and White individuals in the South African cohort (Table 3) and in African and White populations from the International HapMap Project (Table 4). In the South African cohort, the frequency for the CC *Fok*I genotype was significantly higher in Africans compared to Whites (Table 3, *P*<0.050). Similarly, African populations from the International HapMap Project (YRI, LWK and MKK) had a significantly higher frequency for the CC genotype than Whites of Western-European descent (CEU; Table 4, *P*<0.001). Moreover, no significant difference in *Fok*I genotype distribution was observed between YRI, LWK and MKK (Table 4).

**Table 3:** **Frequency distribution of genotypes for the** ***Fok*** **I SNP differs between Africans and Whites from the South African study population.**

Ethnicity	Genotype frequency distribution n (%)		*χ^2^*	*df*	*P*-value
	CC	CT/TT			
African	26 (68.4)	12 (31.4)	5.182	1	<0.050
White	7 (36.8)	12 (63.2)			

**Table 4:** **Frequency distribution of genotypes for the** ***Fok*** **I SNP differs between Africans and Whites, but not between Africans, from the International HapMap Project.**

Ethnicity a	Genotype frequency distribution n (%)		*χ^2^*	*df*	*P*-value
	CC	CT/TT			
YRICEU	73 (65.2)42 (37.2)	39 (34.8)71 (62.8)	17.6	1	<0.001
LWKCEU	62 (72.1)42 (37.2)	24 (27.9)71 (62.8)	22.6	1	<0.001
MKKCEU	88 (62.0)42 (37.2)	54 (38.0)71 (62.8)	12.2	1	<0.001
YRILWKMKK	73 (65.2)62 (72.0)88 (62.0)	39 (34.8)24 (27.9)54 (38.0)	2.456	2	>0.050

a HapMap population data were obtained from the International HapMap Project, including all HapMap Phase I, II and III samples from four populations: Individuals from the Centre d′Etude du Polymorphisme Humain (CEPH) collected in Utah, USA, with ancestry from northern and western Europe (CEU, n = 113); Yoruba in Ibadan, Nigeria (YRI, n = 112); Luhya in Webuye, Kenya (LWK, n = 86) and Maasai in Kinyawa, Kenya (MKK, n = 142).

### *Fok*I influences VDR function, but not expression

**Figure 6:**
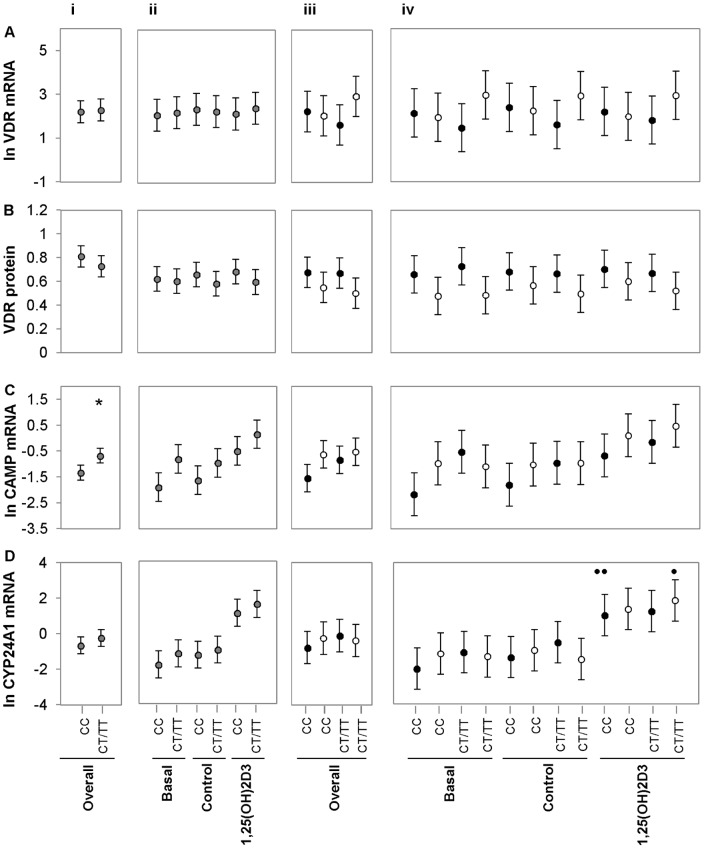
*Fok*I influence VDR function, but not expression. Error-bar plots illustrate the mean level of *VDR* expression (A), VDR protein (B), *CAMP* expression (C) and *CYP24A1* expression (D) differentiated by *Fok*I genotype (CC and CT/TT). Data was analysed combining ethnicity (i and ii, grey dots, n = 57) or differentiating ethnicity (iii and iv, Africans [n = 38], black dots and Whites [n = 19], white dots). Data was further analysed combining (overall, i and iii) or separating treatments (ii and iv). *VDR* expression and protein level was not significantly influenced by *Fok*I genotype. Combining ethnicity and treatment (overall), *CAMP* mRNA level was significantly higher in the CT/TT genotypes compared to the CC genotype (* *P*<0.050). 1,25(OH)_2_D_3_-elicited induction of *CYP24A1* mRNA was significant in Africans with the CC genotype (*P*<0.010, n = 26) and in Whites with the CT/TT genotypes (*P*<0.050, n = 12). Error bars display the LSD for each data set with Bonferroni correction. All significances indicated withstood Bonferroni correction.

To determine whether the *Fok*I genotype influenced *VDR* expression or function, individuals were genotyped for *Fok*I and data analysed based on CC and CT/TT genotype. No significant difference was observed between genotype CC and CT/TT regarding level of *VDR* expression (Fig. 6A) or VDR protein (Fig. 6B) level. The CT/TT genotype showed significantly higher overall levels of *CAMP* mRNA compared to the CC genotype (Fig. 6C i, *P*<0.050). This difference in *CAMP* level between genotypes was not seen at any individual treatment (Fig 6C ii) or between ethnic groups, whether overall (Fig 6C iii) or as defined by treatment (Fig. 6C iv). Furthermore, 1,25(OH)_2_D_3_-elicited induction of *CYP24A1* expression was influenced by *Fok*I genotype, showing interaction with ethnicity (Fig 6D iv). In Africans (Fig 6D iv, black dots), 1,25(OH)_2_D_3_-elicited induction of *CYP24A1* mRNA was significant for only the CC genotype (*P*<0.010, n = 26). In contrast, in Whites (Fig 6D iv. White dots), 1,25(OH)_2_D_3_-elicited induction of *CYP24A1* mRNA was significant with the CT/TT genotype (*P*<0.050, n = 12) but not with the CC genotype (n = 7).

## Discussion

VDR target gene expression is modulated by 1,25(OH)_2_D_3_ [8], [36] and thought to be affected by variant VDR isoforms generated by *Fok*I genotypes [14]. Here we assessed the combined effect of vitamin D, ethnicity and *Fok*I genotype on expression and the functional capabilities of the VDR. Results support that differential VDR expression relates to ethnicity, to a lesser extent to vitamin D status, but not *Fok*I genotype. Instead, VDR transactivation of *CAMP* is influenced by *Fok*I genotype and, together with ethnicity, influence 1,25(OH)_2_D_3_-elicited *CYP24A1* expression. Our results support a complex interaction between *Fok*I, ethnicity and 1,25(OH)_2_D_3_-elicited VDR transactivation capacity of certain target genes, which may explain inconsistent genetic association of *VDR* with disease.

The inconsistent association between *VDR* variants and vitamin D level with diseases in diverse populations led us to investigate the possibility that *VDR* expression, VDR protein level and function may differ between ethnicities. Our results illustrate that ethnicity had a significant main effect on *VDR* expression and protein level, trending towards a higher *VDR* mRNA level in Whites and a significantly higher basal and control VDR protein level in Africans. This inverse relationship between gene expression and protein level suggests differential post-transcriptional regulation between the two ethnicities, and that the dynamics of *VDR* mRNA translation may differ between Africans and Whites. Despite having lower VDR levels than Africans, Whites produced a significantly higher level of *CAMP* mRNA than Africans in response to *in vitro* 1,25(OH)_2_D_3_ supplementation. Increasing the concentrations of *in vitro* 1,25(OH)_2_D_3_ supplementation further illustrated that the response to 1,25(OH)_2_D_3_ supplementation is individual-specific and did not correlate with 25(OH)D_3_ status. While more 1,25(OH)_2_D_3_ seemed beneficial for *CAMP* expression in the Africans that did not respond to 10 nM supplementation, it did not hold any additional benefit for individuals that did. This suggests that optimal 1,25(OH)_2_D_3_ level for VDR function may be individual-specific and that the efficiency of VDR function differs between ethnicities. Furthermore, the relationship between VDR protein level and VDR function observed within these results supports the complex link suggested between regulatory processes and overall phenotype [37]. Ethnicity-dependent VDR activity, as reflected in *CAMP* gene expression, may explain differential disease predisposition, as illustrated by the higher prevalence of TB [38] and colorectal cancer [39] in Africans compared to Whites. Thus, ethnicity influences *VDR* expression, VDR protein level and *CAMP* gene transactivation.

Although target-gene transactivation was directly influenced by availability of 1,25(OH)_2_D_3_ [40], *in vitro* 1,25(OH)_2_D_3_ supplementation had no significant effect on *VDR* expression. While Zella *et al*. (2010) showed that 1,25(OH)_2_D_3_ induced the accumulation of VDR in osteocarcinoma cells [41], Adams *et al*. (2010) and Selvaraj *et al*. (2009), found that neither *in vitro* 25(OH)D_3_ nor 1,25(OH)_2_D_3_ induced *VDR* expression in monocyte-macrophages of healthy individuals, respectively [42], [43]. Combined, the results suggest that while 1,25(OH)_2_D_3_ modulates the innate immune response via the VDR, baseline *VDR* mRNA and protein level are tightly regulated and not influenced by *in vitro* 1,25(OH)_2_D_3_ supplementation or plasma 25(OH)D_3_ level in primary monocyte-macrophages of individuals within normal range for 25(OH)D_3_ status. The effect of 1,25(OH)_2_D_3_ on *VDR* expression and protein level may therefore depend on cell type. Thus, the influence of 1,25(OH)_2_D_3_ on *VDR* expression and VDR protein level in monocyte-macrophages may depend on other confounding factors.

Furthermore, we found that the mean plasma level of 25(OH)D_3_ was not significantly different between Africans and Whites and that both ethnicities had, on average, a normal sufficient 25(OH)D_3_ status according to the Institute of Medicine (IOM) recommendations (>50 nmol/L). In contrast, an American based study found 25(OH)D_3_ status to be lower in Africans than Whites, and that Africans are vitamin D deficient [44], [45]; which may relate to the higher latitude and reduced UVB intensity in North America. A South African study conducted in the Western Cape showed a high prevalence of vitamin D deficiency among Africans, latently infected with *M. tuberculosis* [20]. While latency may influence 25(OH)D_3_ status, vitamin D synthesis is also compromised during the rainy winter months in Cape Town (Latitude 33° S); but not significantly altered throughout the year in individuals living in sunshine-rich Johannesburg (Latitude 26° S) [46]. The lack of disparity in 25(OH)D_3_ status between Africans and Whites in the current study may relate to the higher latitude, Highveld summer rainfall and sunny winter climate. A more recent study conducted in Africa, revealed that the mean serum 25(OH)D concentration of two African populations living in Kenya (Maasai) and Tanzania (Hadzabe) was relatively high. Both populations had normal mean serum 25(OH)D concentrations of 119 nmol/L and 109 nmol/L, respectively [47]. Combined, these findings suggest that 25(OH)D_3_ status is dependent on latitude and climate, with the influence of skin type evident only at less favorable climates. Furthermore, the similar level observed between Africans and Whites in the current study population indicates that, when sufficient, plasma 25(OH)D_3_ status may not be the determining factor in differential *VDR* expression, VDR protein level and function in healthy individuals. The 25(OH)D_3_ status did not correlate with *VDR* expression, VDR protein level or function in our study population. In agreement, Hendrickson *et al*. (2011) and Adams *et al*. (2009) found no association of plasma 25(OH)D_3_ status with *VDR* and *CAMP* expression, respectively [48], [42]. It has been suggested however that plasma 1,25(OH)_2_D_3_ status, independent of 25(OH)D_3_, may differ between individuals. For example, in the case of extra-renal hydroxylation by activated macrophages, where genetic variation in the *VDR* may influence 1,25(OH)_2_D_3_ production and ultimately plasma status [49]. Thus it is possible that 1,25(OH)_2_D_3_ status may have differed between Africans and Whites in the current study and may have influenced differential *VDR* expression, VDR protein level and function.

The genotype distribution of the *Fok*I SNP was significantly different between the two ethnicities in our study, with the CC genotype present in 68% of Africans compared to the 37% in Whites. A similar distribution was observed for HapMap populations. This ethnicity difference in *Fok*I genotype distribution did not appear to influence differential *VDR* expression or VDR protein level between ethnicities. The lack of a significant effect on *VDR* expression and protein level was not entirely unexpected, as *Fok*I is a coding-region SNP which would affect protein function, unlike regulatory-region SNPs. Similarly, Selvaraj *et al*. (2009) found no significant difference in VDR protein level between variant *Fok*I genotypes in both healthy Indian controls and pulmonary TB patients [43]. SNPs in the 3′ end of the *VDR*, together with a variable poly(A) microsatellite have, been shown to influence *VDR* mRNA stability [50], [51]. These variables may be responsible for the inverse relation between *VDR* mRNA and protein level observed in ethnicities of our cohort. Combined, these results suggest that variant genotypes of *Fok*I do not influence *VDR* expression or level, while ethnicity does, implicating environment or other genetic factors.

While the *Fok*I genotype did not affect *VDR* gene regulation in our cohort, it had a target-gene specific effect on VDR function. This finding agreed with the coding, functional nature of *Fok*I; with previous work supporting a more robust transactivation capacity for the shorter VDR isoform (C nucleotide), as shown in *CYP24A1* reporter gene constructs [11], [50], [14]. Van Etten *et al*. (2007) however, found no difference in transactivational capacity between the two alleles using a similar reporter gene assay [13]. In our study, the only evidence for higher activity of the C allele was the significant 1,25(OH)_2_D_3_-dependent induction of *CYP24A1* in Africans for CC homozygotes, but not for those of CT/TT genotype. In contrast, CC homozygosity significantly hampered *CAMP* transactivation, irrespective of ethnicity or condition, and lacked significant 1,25(OH)_2_D_3_-elicited induction of *CYP24A1* in Whites. This suggests that *Fok*I genotype influences VDR transactivation capacity in a target-gene dependent manner. It is possible that the long and short VDR isoforms interact differently with VDRE's in different target genes. This target-gene specific interaction between VDR and VDREs may further be influenced by SNPs in the recognition elements. A low frequency SNP in African-Americans in the VDRE of the *CYP24A1* promoter for example, has been shown to decrease the ability of the VDR to bind to and induce expression of the gene [52]. It is thus possible that VDRE SNPs may differentially influence transactivation of specific target genes. Although the cohort we investigated is small, the functional analysis was done under circumstances closer to normal physiology than reporter assays, and the results suggest that *Fok*I not only has a target-gene specific effect on VDR function, but also interacts with ethnicity and 1,25(OH)_2_D_3_.

Considering that the ancestral allele of *Fok*I is the T nucleotide [53], we propose that the interaction between genotype and ethnicity regarding 1,25(OH)_2_D_3_-elicited induction of *CYP24A1* reflects natural selection. As early homonins with dark pigmented skin migrated North, their pigmentation decreased as an adaptation to synthesize sufficient vitamin D at higher latitude and limited UVB radiation. Modern humans in middle and East Africa however, were exposed to high intensity UV radiation and adapted by increasing skin pigmentation [54]. Despite their dark skin, individuals in Central and East Africa may still produce relatively high levels of 25(OH)D [45]. The more active 1,25(OH)_2_D_3_ breakdown in Africans, facilitated by *CYP24A1*, to regulate high baseline 25(OH)D level provides some support for this hypothesis. Thus, natural selection of the CC genotype of the *VDR*, associated with reduced *CAMP* induction and increased 1,25(OH)_2_D_3_-elicited *CYP24A1* transactivation, was favoured in Africans. Unfortunately, CC likely conferred increased susceptibility to infection with pathogens such as *Mycobacterium tuberculosis*, brought to Africa in the late 1600's to early 1700's through colonisation and trade with the East [55]. Decreased *CAMP* expression in Africans may contribute to the infectious disease burden of South Africa; currently ranked globally with the third highest TB burden [56]. Based on the fact that *CAMP* is a 1,25(OH)_2_D_3_-inducible gene, it is expected that 1,25(OH)_2_D_3_ supplementation would be beneficial in TB prevention and treatment. However, our results suggest that the CC genotype associated with Africans may moderate *CAMP* induction through 1,25(OH)_2_D_3_-elicitated *CYP24A1* induction and consequent 1,25(OH)_2_D_3_ catabolism. Thus, the efficacy of 1,25(OH)_2_D_3_-elicitation and subsequent application in therapy should be considered in the context of ethnicity-dependent variables.

Taken together, differential *VDR* expression relates to ethnicity rather than 25(OH)D_3_ status and *Fok*I genotype, while VDR activity, specifically *CAMP* and 1,25(OH)_2_D_3_-elicited *CYP24A1* transactivation relates to *Fok*I genotype, interacting with ethnicity in the latter case. Contrary to conclusions of previous studies that the CC genotype of *Fok*I results in higher VDR transactivation capacity, our data suggests that *Fok*I genotype influences VDR transactivation capacity in a target-gene dependent manner. Although vitamin D is essential for VDR function, it is likely not the sole contributing factor in VDR-related disease susceptibility, as both the level and activity of the VDR differ between populations. Thus, the expression and role of VDR in target gene transactivation is determined not only by genetics, but also by ethnicity and environment involving complex interactions which may confound disease association. With current literature supporting the long-held belief that genetic variants in the VDR-pathway may be key in the association between vitamin D status and disease susceptibility, future work should evaluate the combined contribution of multiple factors, including environment, in various ethnic groups.
